# Characterization and Interpretation of Cd (II) Adsorption by Different Modified Rice Straws under Contrasting Conditions

**DOI:** 10.1038/s41598-019-54337-1

**Published:** 2019-11-28

**Authors:** Shuai Wang, Nan Wang, Kai Yao, Yuchuan Fan, Wanhong Li, Weihua Han, Xinhua Yin, Dianyuan Chen

**Affiliations:** 1 Institute of Agriculture, Jilin Agricultural Science and Technology University, Jilin, 132101 China; 2 Department of Biosystems Engineering and Soil Science, The University of Tennessee, Knoxville, TN 37996 USA; 3 College of Agricultural Sciences, Hainan University, Haikou, 570228 China; 4 Department of Plant Sciences, The University of Tennessee, Jackson, TN 38301 USA

**Keywords:** Ecology, Environmental sciences, Natural hazards

## Abstract

Rice straw can adsorb Cd(II) from wastewater, and modification of rice straw may improve its adsorption efficiency. The rice straw powder (Sp) from the direct pulverization of rice straw was used as the control, the rice straw ash (Sa), biochar (Sa), and modified rice straw (Ms) were prepared by ashing, pyrolysis and citric acid modification, respectively, and all of them were examined as adsorbents for Cd(II) in this study. Batch adsorption experiments were adopted to systematically compare the adsorption capacities of rice straw materials prepared with different modification methods for Cd(II) from aqueous solution under different levels of initial Cd(II) concentration (0–800 mg·L^−1^), temperature (298, 308, and 318 *K*), contact time (0–1440 min), pH value (2–10), and ionic strength (0–0.6 mol·L^−1^). The results indicated that the modification method affected the adsorption of Cd(II) by changing the specific surface area (SSA), Si content, surface morphology, and O-containing functional group of rice straw. Compared with Sp, Ms held more surface O–H, aliphatic and aromatic groups, while Sa had more phenolic, C–O (or C–O–C), and Si–O groups, and Sb held more C–O (or C–O–C) and Si–O groups; besides, Sa, Sb, and Ms had larger SSA than Sp. Adsorption capacity of the four adsorbents for Cd(II) increased and gradually became saturated with the increase in the initial Cd(II) concentration (0–800 mg·L^−1^). The adsorption capacity of Cd(II) was significantly higher at 318 *K* than 298 *K* and 308 *K*, regardless of the adsorbent type. Sa had the largest SSA (192.38 m^2^·g^−1^) and the largest adsorption capacity for Cd(II). When the initial Cd^2+^ concentration was at 800 mg·L^−1^, the Cd(II) adsorption amount reached as high as 68.7 mg·g^−1^ with Sa at 318 *K*. However, the SSA of Sp was only 1.83 m^2^·g^−1^, and it had the least adsorption capacity for Cd(II). Only the adsorption of Cd(II) upon Sb at 298 *K* was spontaneous, and surprisingly, all other adsorptions were nonspontaneous. These adsorptions were all chemical, and were favorable, exothermic and order-increasing processes. The pseudo-second-order model showed a strong fit to the kinetics of Cd(II) adsorption by the four adsorbents. The adsorption capacities of Cd(II) by the adsorbents were less at low pH, and all were enhanced with the increase of initial pH value (2–10) in the solution. The inhibiting effect on Cd(II) adsorption due to the increase in ionic strength was greater with Sa, Sb, and Ms than that under Sp. The rice straw ash prepared by ashing unexpectedly had greater adsorption capacity for Cd(II) than the biochar and citric acid modified rice straw. The optimum condition for Cd(II) adsorption was established as the temperature of 318 *K*, initial Cd(II) concentration of 800 mg·L^−1^, contact time of 240 min, and no Na(I) interference regardless of absorbent. In conclusion, rice straw ash shows the greatest potential of being applied to paddy fields for the remediation of Cd(II) pollution so as to reduce the risk of Cd(II) enrichment in rice grains and straws.

## Introduction

Cadmium (Cd) is considered one of the most toxic and hazardous heavy metals due to its high mobility and biological accumulation, and can cause a potential threat to human life with prolonged exposure^1–3^. It can be introduced to the aquatic environment through a variety of anthropogenic activities, especially some industrial processes, such as the use of phosphate fertilizers containing Cd and the application of Cd in pigments and stabilizers for plastics^3^.

Rice is the most important food crop in China. As one of the most common agricultural wastes, rice straw can cause many environmental problems since it exists in enormous quantities and is not easy to handle or transport^4^. Direct open burning of rice straw in fields is a common practice for its disposal and causes serious air pollution. Due to the large quantity of hydroxyl, carboxyl, and carbonyl groups, etc. on the surfaces of cellulose, lignin, and hemicelluloses in the rice straw, chelation, ion exchange, and other reactions may occur when rice straw is in contact with heavy metal ions^5^. In addition, because rice straw contains silica, it can adsorb various pollutants from wastewater with high efficiency^6^. Hence, utilization of rice straw to remove Cd(II) from water may be an ideal choice for using waste materials to control pollution^7^.

In order to separate and remove heavy metal pollutants from water bodies, several technologies, such as adsorption, ion exchange, membrane separation, coagulation/flocculation, reverse osmosis, electrodialysis, and chemical precipitation^2^, have been developed in recent years. Out of these technologies, adsorption is considered the most promising method because of its high efficiency, low cost, simple operation, fast response, and environmental friendliness^1,8,9^.

Regeneration, easy access, and low-cost were the criteria used in selecting a suitable adsorbent^10,11^. Raw rice straw could remove Pb(II) from water through biosorption and its maximum removal rate reached 94% under optimal conditions^12^. El-Sayed *et al*.^13^ reported that rice straw was an effective adsorbent for Cd(II), and its adsorption capacity was highly related to the adsorbent dosage, initial Cd(II) concentration, and initial pH. Ding *et al*.^14^ found that the main mechanism of Cd(II) biosorption by raw rice straw lay in the exchange between the Cd(II) pollutant and the K(I), Na(I), Ca(II), and Mg(II) cations in the rice straw, as well as the chelation of Cd(II) by the C = C, C–O, O–H groups and carboxylic acids in the rice straw. Asuquo *et al*.^8^ showed that white yam tuber peels were an efficient adsorbent for removing Cd(II) from aqueous systems.

Chemical pretreatment of agricultural wastes could enhance and reinforce their functional group potential, and consequently increase the adsorption capacity of these biosorbents^15^. Ahmaruzzaman and Gupta^6^ observed that chemical and thermal treatment significantly increased the adsorption capacity of rice husks; however, this increase was dependent on the method and conditions used for the treatment. Ye *et al*.^16^ reported that modified rice husks possessed faster kinetics and greater adsorption capacity for Cd(II) than the raw rice husks owing to the changes in its surface structure. Guo *et al*.^17^ demonstrated that the effects of chemically modified maize straw on Cd(II) removal from aqueous solutions were influenced by adsorbent dosage, initial ion concentration, contact time, solution pH, and temperature. Ong *et al*.^18^ found that the adsorption capacity of rice husks for Cd(II) increased after they were treated with nitric acid. Similarly, the adsorption capacity of rice straw was greatly enhanced through thermochemically modification with citric acid^7^. Wu *et al*.^3^ used the microwave-assisted alkalization and acid oxidation to synthesize two novel wheat straw adsorbents for increasing Cd(II) removal in simulated waterlogged paddy soil. Li *et al*.^19^ utilized three modified biochar materials from rape straw to remediate Cd(II) pollution in aqueous systems. Zhang *et al*.^20^ showed that the biochar modified with H_2_O_2_-HNO_3_ was more effective in removing Cd(II) from water than the rice straw-derived biochar. Feng *et al*.^21^ observed that when rice husks were heated at a high temperature of 700 °C, their adsorption capacity for Pb(II) and Hg(II) from the aqueous solution was enhanced because of the increased specific surface area. Rice husk ash showed significant advantage over rice husks in the removal of Pb(II) from aqueous solution^22^.

In summary, most researchers used only one certain modification method to treat crop straw and explored the reason for the increased adsorption of Cd(II). For crop straws such as rice straw, the differences in adsorption capacity of Cd(II) by crop straw prepared with different modification methods were not simultaneously and systematically compared under conditions with different influencing factors. Thus, the objectives of this research were to (1) examine the effects of different modification methods on rice straw including citric acid modification, ashing and thermal pyrolysis in an oxygen-deficient environment on the characteristics of rice straw adsorbents using scanning electron microscopy (SEM), Fourier transform infrared spectroscopy (FTIR), and Brunauer-Emmett-Teller (BET) specific surface area measurement instrument, and (2) investigate the influences of initial Cd(II) concentration, temperature, contact time, solution pH, and ionic strength on the adsorption capacities of Cd(II) with the rice straw adsorbents and explore their differences in the adsorption amount of Cd(II).

## Materials and methods

### Preparation of rice straw adsorbents

Rice straw (*Oryza sativa* L.) was collected from the experimental station at Jilin Agricultural Science and Technology University, Jilin, China. The rice straw was washed after harvest, rinsed with distilled water, and then air dried for 2 weeks. Preparation of rice straw powder (Sp): The air-dried rice straw was chopped into 10 mm length, directly pulverized with a grinder without any treatment, and passed through a 0.25-mm sieve. Preparation of rice straw ash (Sa): The ground rice straw powder was placed in a thin stainless steel tray, and the bottom of the tray was burned with an alcohol lamp while continuously turning over the straw until fully ashed. Preparation of rice straw biochar (Sb): The chopped rice straw was placed in a 700-mL ceramic crucible, covered with a lid, and pyrolyzed in the absence of oxygen using a muffle furnace. The temperature was raised at 20 °C·min^−1^ and kept constant at 500 °C for 2 h^23^. It was cooled down at room temperature and then ground to pass through a 0.25-mm sieve. Preparation of citric acid modified rice straw (Ms): The rice straw powder was mixed with 0.6 mol·L^−1^ citric acid at a ratio of 1:12 (straw/acid, *w*/*v*); after stirring for 30 min at room temperature, the acidic straw slurries were placed in a stainless-steel tray and dried at 50 °C in a forced-air oven. After 24 h, the thermochemical reaction between acid and straw was followed by raising the oven temperature to 120 °C for 90 min. After cooling, the reacted products were washed with distilled water to remove excess citric acid. Lastly, the washed reacted products were dried until a constant weight was achieved, and were then cooled down in a desiccator^4,7^.

### Characterization of rice straw adsorbents

Specific surface area (SSA) and pore volume of the four adsorbents were estimated using the BET nitrogen adsorption technique at 77.3 *K* in a specific surface and aperture analyzer [3H-2000PS1, Beishide Instrument Technology (Beijing) Co., Ltd., China]; Scanning electron microscope (SEM) imaging analysis was conducted with a field emission scanning electron microscope (Hitachi, SU8000, Tokyo, Japan) to compare the structural and surface characteristics of the four types of rice straw adsorbents, and the scale was 5 µm. For Fourier transform infrared spectroscopy (FTIR), the sample was dried in a vacuum oven at 100  °C for 3 h before measurement. A sample of 1.5 mg was compressed under vacuum with 250 mg of KBr at a pressure of 20 MPa. The pellets obtained were analyzed with an FTIR-850 spectrometer from Gangdong Sci & Tech Development Co, Ltd. in China covering a frequency range of 4000 to 400 cm^−1^.

### Cd(II) adsorption experiments

0.05 g of an adsorbent was accurately weighed into a 50-mL polyvinyl tube, and then a certain concentration of CdCl_2_ and NaCl (a supporting electrolyte) was added. The Cd(II) adsorption experiments consisted of the following four parts. (1) For adsorption thermodynamics, the initial Cd(II) concentration was set at 0, 20, 40, 80, 120, 200, 300, 400, 500, 600 and 800 mg·L^−1^, respectively. The isothermal adsorption experiments were carried out at three temperatures (298 *K*, 308 *K*, and 318 *K*) separately. (2) For adsorption kinetics, the initial Cd(II) concentration was 400 mg·L^−1^, and the suspensions were shaken at an agitation rate of 150 r·min^−1^ at 298 *K*, 308 *K*, and 318 *K* for different contact times of 0, 10, 30, 60, 90, 120, 240, 360, 480, 720, and 1440 min, respectively. (3) For the influence of pH values, the temperature was 298 *K*, the initial Cd(II) concentration was 400 mg·L^−1^, and the initial pH value was set to 2, 3, 4, 5, 6, 7, 8, 9, and 10, respectively, with 0.1 mol·L^−1^ NaOH or HCl. The final pH value was referred to as the pH value of the equilibrium solution at the end of adsorption. (4) For the effect of ionic strengths, the temperature was at 298 *K*, the initial Cd(II) concentration was 400 mg·L^−1^, and the ionic strength (Na(I)) was set to 0, 0.004, 0.01, 0.04, 0.1, 0.2, 0.4, and 0.6 mol·L^−1^, respectively, using NaCl solution. The samples from each experiment were repeated three times.

The total volume of equilibrium solution was 25 mL, and the adsorption experiments were carried out in a constant temperature water bath shaker for a predetermined time, shaking for 10 h and resting for 14 h, except for adsorption kinetics. Then, the centrifuge tubes were taken out, centrifuged at 12,000 r·min^−1^ for 10 min, filtered, and diluted to a certain Cd(II) concentration, which was determined with an atomic absorption spectrophotometer (TAS 990) produced by Beijing Puxi General Co., Ltd. in Beijing, China. The amount of adsorbed Cd(II) at equilibrium (*q*_*e*_, mg·g^−1^) was calculated using the following equation (Eq. (1)):

1 $${q}_{e}=({C}_{0}-{C}_{e})V/{m}_{s}$$

where *C*_0_ and *C*_*e*_ (mg·L^−1^) were the initial and equilibrium concentrations of Cd(II), respectively. *V* (*L*) was the volume of the solution, and *m*_s_ (g) was the mass of adsorbent. All experiments were conducted with three replicates at room temperature. The Cd(II) concentration was reported as an average of the three replicates. The adsorption isotherm was modeled with the Langmuir (Eq. (2)), Freundlich (Eq. (3)) and Temkin (Eq. (4)) isotherm models, and the adsorption kinetics were simulated using the Lagergren’s pseudo-first order (Eq. (5)) and pseudo-second-order (Eq. (6)) kinetic equations. All the data were calculated using the Origin 8.0 software.

2 $${q}_{e}=\frac{{q}_{m}{K}_{L}{C}_{e}}{1+{K}_{L}{C}_{{\rm{e}}}}$$

3 $${q}_{e}={K}_{F}{C}_{e}^{1/n}$$

4 $${q}_{e}={B}_{1}\,\mathrm{ln}({K}_{T}{C}_{e})$$

5 $${q}_{t}={q}_{e}(1-{e}^{-{k}_{1}t})$$

6 $${q}_{t}=\frac{{k}_{2}{q}_{e}^{2}t}{1+{k}_{2}{q}_{e}t}$$

where *q*_*e*_ and *q*_t_ (mg·g^−1^) were the adsorption capacity at equilibrium and time *t*, respectively; *k*_1_ (min^−1^) and *k*_2_ (g·mg^−1^·min^−1^) were the rate constants corresponding to the respective kinetic model; *C*_*e*_ (mg·L^−1^) was the residual Cd(II) concentration in the solution at equilibrium; *q*_*m*_ (mg·g^−1^) represented the maximum adsorption capacity; *K*_*L*_ (L·mg^−1^) was the Langmuir adsorption constant related to the free energy of adsorption; *K*_*F*_ (mg·g^−1^) was a constant in the Freundlich model relating to the adsorption capacity; and 1/*n* was an empirical parameter relating to the adsorption intensity, which varied with the heterogeneity of the material. *B*_1_ was the Temkin constant related to the heat of adsorption (kJ·mol^−1^), and *K*_*T*_ (L·mg^−1^) was the equilibrium binding constant relating to the maximum binding energy^8^.

For the Langmuir model, to determine whether the adsorption system was favorable or not, the isotherm was classified with the separation factor (*R*_*L*_), which was calculated with the following equation (Eq. (7))^24,25^:

7 $${R}_{L}=\frac{1}{1+{K}_{L}{C}_{0}}$$

To describe the thermodynamic behavior of the adsorption of Cd(II) onto the four rice straw biosorbents, thermodynamic parameters, such as Enthalpy change (Δ*H*°), Gibbs free energy change (Δ*G*°) and Entropy change (Δ*S*°) were estimated. The thermodynamic parameters of the sorption reaction were assessed with the following equations (Eq. (8) and Eq. (9))^26^:

8 $$\Delta G^\circ =-\,RT\,\mathrm{ln}({K}_{L})$$

9 $$\Delta G^\circ =\Delta H^\circ -T\Delta S^\circ $$

where *T* was thermodynamic temperature (*K*); *R* was the gas constant (8.314 J·mol^−1^·*K*^−1^); and *K*_*L*_ was the equilibrium constant obtained from the Langmuir isotherm.

## Results and discussion

### Characterization of the adsorbents

The N_2_-BET specific surface area (SSA) was 1.83, 192.38, 16.47, and 3.17 m^2^·g^−1^ and the pore volume was 0.016, 0.228, 0.045, and 0.020 cm^3^·g^−1^ for Sp, Sa, Sb, and Ms, respectively. Ahmaruzzaman and Gupta^6^ showed that the modification of rice husks could remove lignin and hemicellulose, reduce cellulose crystallinity, and increase the porosity or surface area. The SSA and pore volume results presented the same trend in our study, indicating that the microstructure and pore properties of rice straw benefited from the modification^27^. The Tiron-extractable Si contents were 41.2, 182.3, 168.5, and 20.1 g·kg^−1^ in Sp, Sa, Sb, and Ms, respectively, according to the analytical method of Guntzer *et al*.^28^.

**Figure 1:**
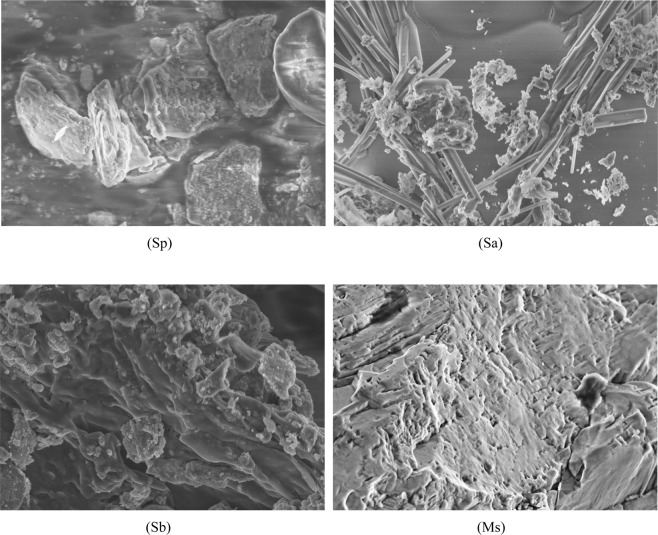
SEM images of the four rice straw adsorbents at 10,000x resolution (5 μm). Sp, rice straw powder; Sa, rice straw ash; Sb, rice straw biochar; and Ms, modified rice straw.

The SEM image of Sp in Fig. 1 (Sp) showed Sp had a granular structure with irregular plates and cracks on its coarse surface^6,25^. The SEM image of Sa in Fig. 1 (Sa) showed that Sa had a large number of needle structures. The SEM image of Sb in Fig. 1 (Sb) showed there were many wrinkles on the surface of Sb, and its edges had more granular structures. The SEM image of Ms in Fig. 1 (Ms) showed that Ms had a porous structure. It was obvious that different modification methods exerted different effects on the surface morphology of rice straw, which might affect the removal of Cd(II) by rice straw from water.

**Figure 2:**
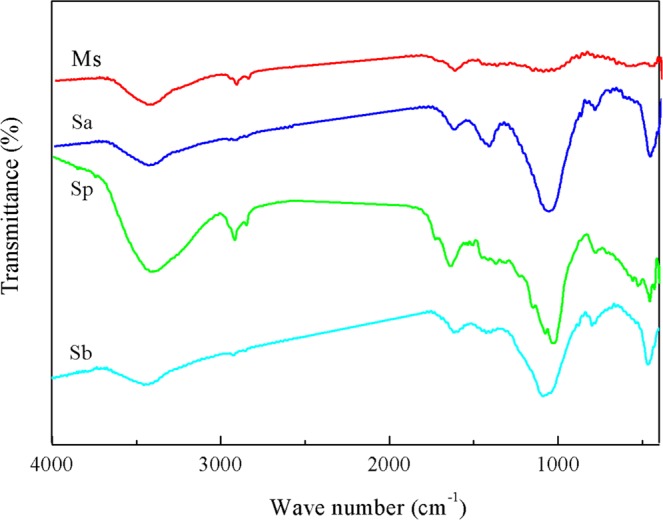
FTIR spectra of the four rice straw adsorbents. Sp, rice straw powder; Sa, rice straw ash; Sb, rice straw biochar; Ms, modified rice straw.

The FTIR spectra of four rice straw adsorbents were presented in Fig. 2. The characteristic peaks’ position and spectral assignment for the adsorbents were shown in Table 1. As could be seen from Fig. 2, the FTIR spectra of four adsorbents had characteristic peaks at 3404~3454 cm^−1^ and 2924~2852 cm^−1^, which could be attributed to the O–H stretching vibration corresponding to the aliphatic moieties in lignin and polysaccharides (cellulose and hemicellulose) and saturated C–H stretching vibration (-CH_2_ and -CH_3_) in cellulose^3^. Compared with Sp, Ms had more surface O–H groups and aliphatic groups (-CH_2_ and -CH_3_), but the proportions of these groups were lower in Sa and Sb. Both incineration and pyrolysis (Sa and Sb) could remove large amounts of cellulose and hemicellulose from Sp^6,25^ through depolymerization and decomposition, correspondingly reducing the surface O-H and aliphatic groups. The peak at 1734 cm^−1^ was attributed to unionized C = O stretching of carboxylic acid, which was only observed in Sp. The band at 1616~1647 cm^−1^ was assigned to stretching in aromatic rings from lignin^8^. Relative to Sp, Ms also had an advantage in terms of aromaticity, and incineration and pyrolysis (Sa and Sb) eliminated a part of lignin and weakened the aromaticity of Sp. The above changes in the functional groups of Sa and Sb relative to Sp were all similar to those of Park *et al*.^29^. The peak observed at 1373~1419 cm^−1^ corresponded to the aliphatic deformation of CH_2_ or CH_3_ group or O–H group of phenols^19,29,30^. Park *et al*.^29^ also concluded that this peak would disappear in Ms after the modification with citric acid. Incineration of rice straw (Sa) was more conducive to the increase of these functional groups. The peak at 1036~1088 cm^−1^ was attributed to the stretching vibration of C–O or C–O–C in cellulose and hemicelluloses^31,32^. Both Sa and Sb had greater advantages over Sp in terms of the amount of functional groups. These functional groups had the ability to some extent to bind Cd(II) by donation of an electron pair from these groups to form complexes with Cd(II) in solution^33^. The absorption bands at 793~798 cm^−1^ and 461~467 cm^−1^ were related to Si–O vibrations, in accordance with the siliceous nature of the ashes ^31^. The peak at 461~467 cm^−1^ was attributed to Si–O bending vibration, indicating of the silica presence^34^. There was no peak at 461~467 cm^−1^ in Ms, indicating that a portion of silica was removed from rice straw after its modification with citric acid. Our result is consistent with the conclusion of Rocha *et al*.^34^ However, incineration and pyrolysis (Sa and Sb) greatly reduced the volume of Sp, and thus making the Si content concentrated and increased in Sa and Sb. The ordering of the Si content in the four adsorbents was also indirectly reflected in the FTIR spectra, which is in agreement with the actual Si measurement results. It could be inferred that compared with Sp, Ms hold more surface O–H groups, aliphatic groups (-CH_2_ and -CH_3_) and aromatic rings after the modification with citric acid, while Sa and Sb had more phenolic, C–O or C–O–C, and Si–O groups. The characteristics of these functional groups all contributed to the increase in the adsorption capacity of Cd(II).

**Table 1:** Relative intensities (%) of the characteristic peaks existed in the four rice straw adsorbents.

Treatments										
Ms	Positions (cm^−1^)	3423	2924	2852		1630		1074		
	Relative intensities (%)	61.93	2.78	0.49		9.31		25.49		
Sa	Positions (cm^−1^)	3438	2920	2858		1630	1419	1066	793	463
	Relative intensities (%)	18.73	0.38	0.09		2.90	5.43	58.30	1.74	12.43
Sp	Positions (cm^−1^)	3404	2922	2852	1734	1647	1373	1036		461
	Relative intensities (%)	50.15	2.14	0.10	0.10	5.23	3.42	38.66		0.20
Sb	Positions (cm^−1^)	3454	2924	2854		1616	1398	1088	798	467
	Relative intensities (%)	22.83	0.35	0.15		3.99	1.43	58.40	3.17	9.67

Sp, rice straw powder; Sa, rice straw ash; Sb, rice straw biochar; Ms, modified rice straw.

### Adsorption isotherms

**Figure 3:**
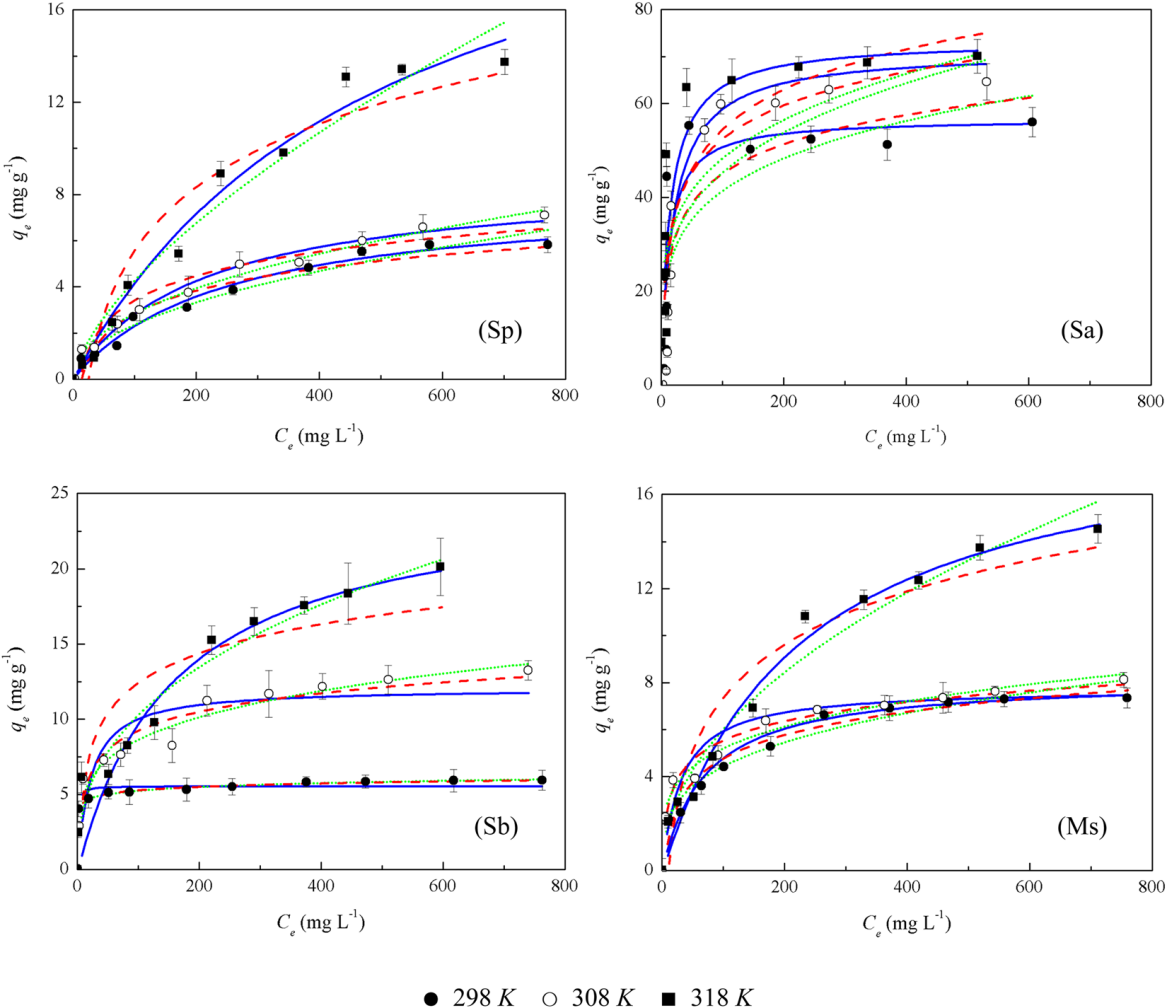
Effect of initial Cd(II) concentration and temperature on the adsorption of Cd(II) onto four rice straw adsorbents. The fitted results of the Langmuir, Freundlich and Temkin models were represented by a solid, short dot, and dash line, respectively. Sp, rice straw powder; Sa, rice straw ash; Sb, rice straw biochar; Ms, modified rice straw.

The reaction temperature of the system plays an important role in adsorption capacity^6^. The adsorption isotherm results are presented in Fig. 3. The results demonstrated that the adsorption capacity of Cd(II) with the four adsorbents increased until the Cd(II) adsorption reached the state of equilibrium and saturation. The initial Cd(II) concentration in the solution provided an important driving force to overcome all mass-transfer resistance for Cd(II) ions between the aqueous and solid phases^6^. The collision efficiency between Cd(II) and the adsorbent increased with the increase in the initial Cd(II) concentration in the solution. The increase in adsorption capacity was due to the increase in the driving force of Cd(II) to the adsorption sites^35^. However, the adsorption capacity of Cd(II) was no longer enhanced and remained almost constant after saturation. This was due to the lack of binding sites on the adsorbent for adsorbing Cd(II) at higher Cd(II) concentrations^33^. When Sp and Ms were used as the adsorbents, there was no obvious difference between the adsorption capacities of Cd(II) at 298 *K* and 308 *K*, but the highest temperature at 318 *K* resulted in greater adsorption capacity than the two lower temperatures. However, when Sa and Sb were utilized as the adsorbents, the adsorption capacity of Cd(II) increased gradually with the increase in temperature from 298 *K* to 318 *K*. The adsorption of Cd(II) onto the adsorbent with a larger SSA was more sensitive to the temperature than that with a smaller SSA. A higher SSA of an adsorbent indicated more adsorption sites, contact area and surface functional groups^27^. Therefore, the adsorbent with a higher SSA was more susceptible to temperature change and thus the thermal motion of the molecule. Increased temperature could increase the mobility of Cd(II) and produce a swelling effect within the internal structure of the adsorbent enabling Cd(II) to penetrate further^36^.

The adsorption capacity of Cd(II) at 298 *K* had the following order: Sa > Sb≈Ms > Sp. Specifically, when the initial Cd(II) concentration was 0~120 mg·L^−1^, the adsorption capacity for Cd(II) was greater with Ms than Sb, but when the initial Cd(II) concentration was in the range of 120~800 mg·L^−1^, Sb had a higher adsorption capacity than Ms. Different from the above trends, the adsorption capacities of the four adsorbents for Cd(II) at 308 *K* and 318 *K* followed the order: Sa > Sb > Ms > Sp. This is consistent with the order of the SSA of four adsorbents, which could reflect the adsorption capacities of the adsorbents indirectly. A larger SSA meant more adsorption sites, i.e., greater adsorption capacity^37^. This is similar to the report by Wu *et al*.^3^. Although the SSAs of Ms and Sp were almost equal, the introduced, free carboxyl groups from citric acid could increase the net negative charges on the rice straw fiber, thereby increasing its adsorption capacity for Cd(II)^7^. When the temperature was set at 318 *K* and the initial concentration Cd(II) was set to 800 mg·L^−1^, the adsorption amount of Cd(II) by Sa reached 68.7 mg·g^−1^. This was the highest adsorption value out of the four rice straw adsorbents, which was much higher than the results of forest biowastes, but still less than that from the cashew nutshell treated with H_2_SO_4_ (436.7 mg·g^−1^) and the result from orange peel modified with KCl (125.6 mg·g^−1^)^15^.

Finally, these results were fitted with the Langmuir, Freundlich, and Temkin isotherm models, and the adsorption constants and correlation coefficients derived from the isotherms at different temperatures are presented in Table 2. Comparing the mean values of *R*^2^ at the three different temperatures for each fitting equation under the same adsorbent, the fitting effects of Langmuir and Freundlich equations were better than those of the Temkin equation on Sp; the fitting effects of Langmuir and Temkin equations were better than those of the Freundlich equation on Sa; and the fitting effects of Freundlich and Temkin equations were better than those of the Langmuir equation on Sb and Ms. It could be clearly seen that the maximum adsorption capacity (*q*_*m*_) of the Langmuir equation for the four adsorbents was greater at 318 *K* than 298 *K* and 308 *K*. The *K*_*F*_ value in the Freundlich equation was a measure of the degree of adsorption. The higher *K*_*F*_ values at lower temperatures indicated that more sorption would be expected at these temperatures^38^. As shown in Fig. 3, the *K*_*F*_ value of Cd(II) adsorbed by Sb was the largest at 298 *K* out of the three temperatures, indicating more sorption of Cd(II) on the rice straw biochar would be expected at 298 *K*.

**Table 2:** Adsorption equilibrium constants obtained from Langmuir, Freundlich and Temkin isotherms in the adsorption of Cd(II) onto the four rice straw adsorbents.

Treatments	Temperature (*K*)	Langmuir			Freundlich			Temkin		
		*q*_*m*_ (mg g^−1^)	*K*_*L*_ (L mg^−1^)	*R*^2^	*K*_*F*_ (mg^(1-1/n)^ g^−1^ L^1/n^)	*1/n*	*R*^2^	*B*_*1*_	*K*_*T*_	*R*^2^
Sp	298	8.00334	0.00403	0.97041	0.24775	0.49038	0.95810	1.41157	0.07457	0.93204
	308	8.76246	0.00467	0.96685	0.34308	0.46124	0.98589	1.51178	0.09591	0.94104
	318	25.2405	0.00199	0.97643	0.20699	0.65831	0.95180	3.96250	0.04058	0.92862
Sa	298	56.73956	0.0833	0.72283	15.22602	0.21885	0.56832	9.00016	1.47786	0.72848
	308	70.04386	0.04055	0.91422	11.74713	0.29859	0.74798	13.86945	0.40946	0.89936
	318	72.80991	0.05567	0.77991	14.82427	0.26600	0.65398	12.89211	0.73158	0.78507
Sb	298	5.5494	1.18848	0.62414	3.88294	0.06518	0.98366	0.32618	106895.0	0.99777
	308	12.08095	0.04896	0.76533	2.99239	0.22961	0.94509	1.76692	1.87956	0.95812
	318	25.1658	0.00632	0.88818	1.71175	0.38917	0.94648	2.77699	0.88637	0.85144
Ms	298	8.14353	0.0142	0.94030	1.12171	0.29845	0.94945	1.42423	0.28727	0.96985
	308	7.7523	0.03237	0.84066	1.76356	0.23455	0.96661	1.17731	1.10837	0.97634
	318	19.32293	0.00446	0.97593	0.63067	0.48946	0.96220	3.27095	0.09383	0.92453

Sp, rice straw powder; Sa, rice straw ash; Sb, rice straw biochar; Ms, modified rice straw.

All values of 1/n less than 1 in Table 2 indicated the favorable nature of adsorption of Cd(II) onto the four rice straw adsorbents^25^, and the adsorption capacity was only slightly suppressed at a lower initial concentration of Cd(II). This isotherm did not predict any saturation of Cd(II) by the adsorbent; thus, infinite surface coverage was predicted mathematically, indicating multilayer adsorption of Cd(II) on the surfaces of rice straw adsorbents^39^.

The *R*_*L*_ and thermodynamic parameters were calculated according to Eqs. (7)-(9) and are presented in Table 3. The *R*_*L*_ parameter gave an important sign for the possibility that the adsorption process might be irreversible (*R*_*L*_ = 0), favorable (0 < *R*_*L*_ < 1), linearly adsorbed (*R*_*L*_ = 1) or unfavorable (*R*_*L*_ > 1)^24^. It can be seen from Table 3 that the *R*_*L*_ values in Cd(II) adsorption of the four adsorbents was in the range of 0~1, indicating that the adsorption was a favorable process. This is consistent with the findings of Ding *et al*.^14^. A negative value for Δ*G*° occurred only when Cd(II) was adsorbed by Sb at 298 *K*, indicating the adsorption process of Cd(II) onto the rice straw biochar at 298 *K* was thermodynamically feasible and of spontaneous nature. However, all other Δ*G*°s were positive, suggesting the nonspontaneous nature of the adsorption process. It was meant an energy barrier existed during adsorption^40^. The nonspontaneous nature of Cd(II) adsorption in our study is similar to that reported by Albadarin *et al*.^41^. The negative values of Δ*H*° for Cd(II) adsorption onto the four adsorbents confirmed the exothermic nature of the adsorption process^13,38^, and the negative Δ*S*° values reflected the order of adsorption increased during the adsorption process^40^.

**Table 3:** Thermodynamic parameters for the adsorption of Cd(II) onto the four rice straw adsorbents.

Treatments	Temperature (*K*)	Δ*G*° (kJ g^−1^ mol^−1^)	Δ*H*° (J g^−1^ mol^−1^)	Δ*S*° (J g^−1^ mol^−1^ *K*^−1^)	*R*_*L*_
Sp	298	13.66	−27.76	−0.14	0.1988~1
	308	13.74			0.1764~1
	318	16.44			0.3344~1
Sa	298	6.16	−15.89	−0.07	0.0119~1
	308	8.21			0.0241~1
	318	7.64			0.0176~1
Sb	298	−0.43	−206.35	−0.69	0.0008~1
	308	7.73			0.0200~1
	318	13.39			0.1366~1
Ms	298	10.54	−45.63	−0.19	0.0658~1
	308	8.78			0.0300~1
	318	14.31			0.1832~1

Sp, rice straw powder; Sa, rice straw ash; Sb, rice straw biochar; Ms, modified rice straw.

### Adsorption kinetics

**Figure 4:**
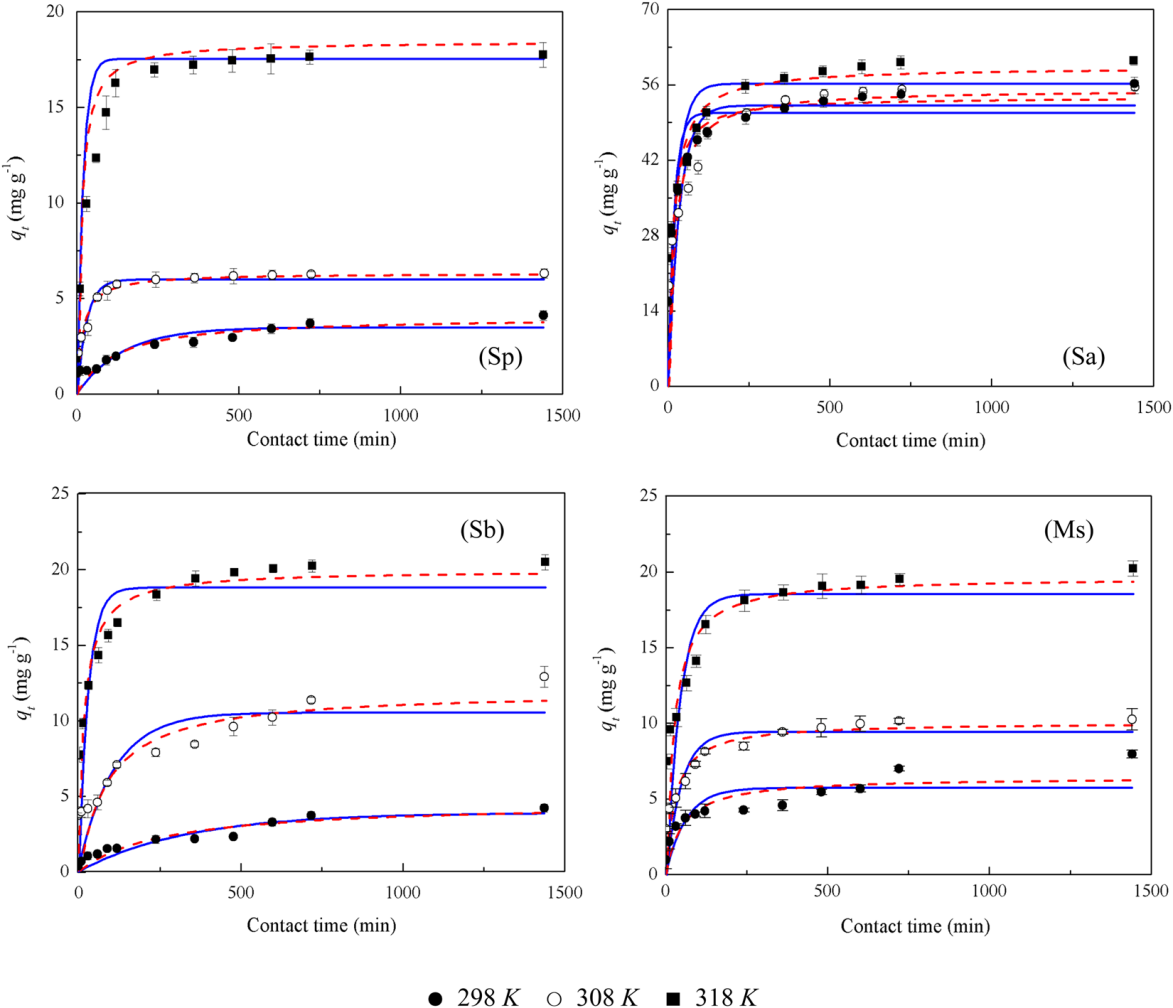
Effect of contact time on the adsorption of Cd(II) onto the four rice straw adsorbents. The fitted results of the Lagergren’s pseudo-first-order and pseudo-second-order models are represented by a solid and short dot line, respectively. Sp, rice straw powder; Sa, rice straw ash; Sb, rice straw biochar; Ms, modified rice straw.

The Cd(II) adsorption capacities of the four adsorbents increased with contact time and then stabilized when an equilibrium was reached (Fig. 4). During the first 240 min, the adsorption rate of Cd(II) with the four adsorbents reached more than 50%, and as the temperature increased, the adsorption rate of each adsorbent was enhanced.

The adsorption of Cd(II) by rice straw is a rapid process^14^. Figure 4 presents the kinetics of Cd(II) adsorption onto the four adsorbents. The adsorption capacities of these four adsorbents increased with contact time and then stabilized when an equilibrium was reached. This is consistent with the findings of Zhang *et al*.^20^. During the first 240 min, Cd(II) in the solution was rapidly adsorbed by the four adsorbents. The adsorption capacity of Cd(II) by Sp accounted for 63.2%, 94.8%, and 97.0% of the final adsorption rate at the contact time of 1440 min under 298, 308 and 318 *K*, respectively. Similarly, the adsorption rates were 88.8%, 91.3%, and 92.1% for Sa, 50.3%, 61.0%, and 89.5% for Sb, and 53.2%, 82.6%, and 89.5% for Ms of the final adsorption rate at 1440 min under 298, 308 and 318 *K*, respectively. It was seen that in the first 240 min, the adsorption of Cd(II) by the four adsorbents reached more than 50% of the final adsorption capacity regardless of temperature; as the temperature was raised, the adsorption capacity accounting for the percentage of the final adsorption capacity increased. Considering practical operation, the optimum contact time was selected as 240 min. The rapid adsorption during the first 240 min could be attributed to the availability of the abundant functional groups and empty adsorption sites on the adsorbent^42^; while with increase in contact time, the remaining vacant surface adsorption sites were difficult to be occupied due to repulsive forces between the solute molecules on the solid phases and slow pore diffusion or saturation of the adsorbent^25^.

Oeverall, Sb showed significant advantages in Cd(II) adsorption over the other three adsorbents in our study. To investigate the possible mechanism of adsorption, pseudo-first-order and pseudo-second-order adsorption models were used to fit the data. The kinetic results for the adsorption of Cd(II) by the four adsorbents are given in Table 4. The low correlation coefficient values (*R*^2^) at all temperatures suggest that the pseudo-first-order kinetic model be not suitable for describing the kinetics of the adsorption process. In contrast, the pseudo-second-order kinetic model showed strong fit to the data in our study, indicating that the adsorption process was chemical adsorption involving valence forces through the exchange or sharing of electrons between Cd(II) and the adsorbents^17,25^.

**Table 4:** Kinetic parameters obtained from kinetic models for the adsorption of Cd(II) onto the four rice straw adsorbents.

Treatments	Temperature (*K*)	Lagergren’s pseudo-first order			Pseudo-second-order		
		*q*_*t*_ (mg g^−1^)	*k*_*1*_ (min^−1^)	*R*^2^	*q*_*t*_ (mg g^−1^)	*k*_2_ (g mg^−1^ min^−1^)	*R*^2^
Sp	298	3.4924	0.0071	0.6975	4.0576	0.0021	0.7732
	308	6.0563	0.0348	0.6893	6.3764	0.0097	0.7482
	318	17.5582	0.0561	0.5047	18.4610	0.0050	0.6021
Sa	298	50.7966	0.0529	0.6981	53.7478	0.0015	0.8038
	308	52.1946	0.0290	0.5632	55.0369	0.0010	0.7005
	318	56.2693	0.0356	0.4534	59.5281	0.0010	0.5978
Sb	298	3.9211	0.0009	0.8395	4.5635	0.0008	0.8805
	308	10.4265	0.0093	0.6137	11.9465	0.0010	0.7123
	318	18.7622	0.0123	0.4801	19.9148	0.0029	0.6300
Ms	298	5.7334	0.0161	0.6294	6.4436	0.0031	0.7537
	308	9.5123	0.0212	0.6846	10.1284	0.0036	0.7831
	318	18.6134	0.0239	0.4961	19.6157	0.0023	0.6240

Sp, rice straw powder; Sa, rice straw ash; Sb, rice straw biochar; Ms, modified rice straw.

### Effects of pH

**Figure 5:**
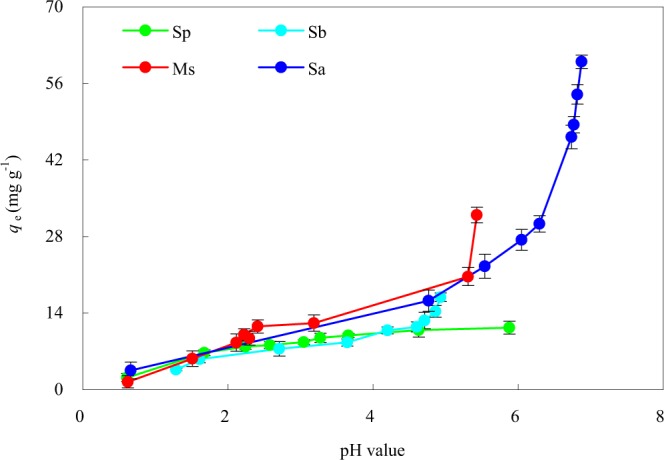
Effects of final pH on the adsorption of Cd(II) onto the four rice straw adsorbents. Sp, rice straw powder; Sa, rice straw ash; Sb, rice straw biochar; Ms, modified rice straw.

The pH value plays a decisive role in heavy metal ion biosorption^26^. Figure 5 shows the effect of the final pH value on the Cd(II) adsorption capacities of the four adsorbents. These final pH values were obtained at the initial Cd(II) concentration of 400 mg·L^−1^ and initial pH value ranging from 2 to 10. These results showed that the Cd(II) adsorption capacities of the four adsorbents were less at low pH values. The following mechanisms could explain this phenomenon. Firstly, the protonation of functional groups in the adsorbents made the number of available functional groups decreased. There existed electrostatic repulsion between the protonated functional groups and the positively charged Cd(II)^14^, which could impede the reaction of the adsorbents with Cd(II)^26^. Secondly, the surface active adsorption sites of the adsorbents were occupied by H(I) and H_3_O(I), which prevented Cd(II) from approaching the adsorption sites on the adsorbents^43^.

The adsorption capacity of Cd(II) increased with the increase of initial pH in the solution regardless of adsorbent type. At higher pH values, the lower number of H(I) and a greater number of ligands with negative charges resulted in greater Cd(II) biosorption^33^. When there were more negative charges on the adsorbent’s surface, the electrostatic interactions were enhanced between the adsorbent and Cd(II)^44^, and the adsorption of Cd(II) was enhanced^40^. Another aspect that should be considered was the metal speciation in solution which was also pH-dependent. An increase in solution pH resulted in further hydrolysis of Cd(II)^45^, in which several low-soluble hydroxyl complexes such as Cd(OH)_2_ and Cd(OH)_3_^−^ might be formed^46^. Thus, the maximum Cd(II) adsorption capacities of 11.3, 60.0, 16.9 and 31.9 mg·g^−1^ were obtained for Sp, Sa, Sb, and Ms, respectively, at the greatest pH value.

After Cd(II) adsorption, the final pH values of the equilibrium solutions from Sp, Sa, and Sb to Ms were decreased to 5.88, 6.88, 4.93, and 5.43, respectively. Obviously, more organic functional groups with H(I) in the adsorbents participated in the exchange reaction. It led to more H(I) entering the solution and buffering the increase in pH value^26^. In modification of Sp to Sb, the buffer capacity to alkali was maximized. This was due to that fact that the preparation of biochar (Sb) produced organic acids and phenolic substances that lowered the pH in the equilibrium solution^47^. However, Sa had a relatively weak buffering capacity for alkali, owing to its higher Ca and Mg contents^48^, so the Cd(II) adsorption capacity was greatly improved in Sa with the increase in solution pH.

### Effects of ionic strength

**Figure 6:**
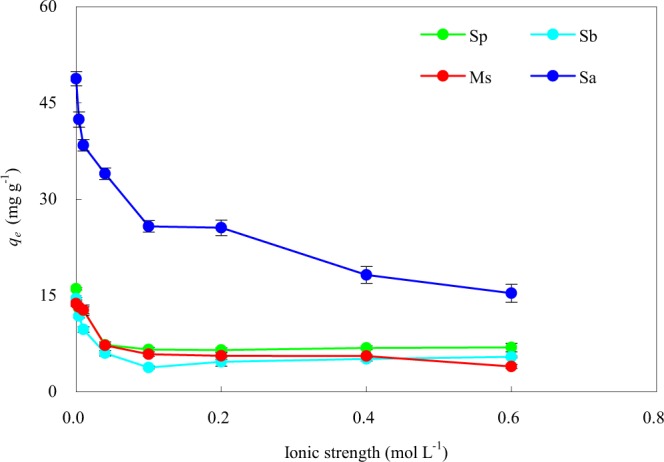
Effect of initial ionic strength on the adsorption of Cd(II) onto the four rice straw adsorbents. Sp, rice straw powder; Sa, rice straw ash; Sb, rice straw biochar; Ms, modified rice straw.

The effect of ionic strength on Cd(II) adsorption on the four adsorbents was achieved and examined through varying the concentrations of additive NaCl from 0 to 0.6 mol·L^−1^. As shown in Fig. 6, the adsorption of Cd(II) onto the four adsorbents was obviously affected by ionic strength. The Cd(II) adsorption with Sa and Ms decreased gradually with an increase in NaCl concentration. A reasonable explanation lay in that there was competitive adsorption between Cd(II) and Na(I); in other words, Na(I) competed with Cd(II) for the adsorption sites during the sorption process under the four adsorbents^49^. At a lower ionic strength, more functional groups from Sa and Ms were available for Cd(II) adsorption, thus the effect of Na(I) on Cd(II) adsorption was insignificant. However, when the ionic strength was higher, the competition between Cd(II) and Na(I) for the available adsorption sites became more important^50^; enhanced competition of the background electrolyte cation (Na(I)) for deprotonated adsorption sites at higher ionic strength^51^ resulted in lower Cd(II) adsorption. Similar results were reported by Chen *et al*.^52^ for Cd(II) adsorption by bentonite. But for Sp and Sb, as the ionic strength (Na(I) concentration) increased, the adsorption capacities of Cd(II) decreased first and then increased slightly. The inhibiting effect of lower Na(I) concentrations on Cd(II) adsorption was explained by the competition between Na(I) and Cd(II) for the limited adsorption sites on the surfaces of adsorbents^53^. However, a higher concentration of Na(I) (0.4~0.6 mol·L^−1^) could not completely occupy the active adsorption sites of Sp and Sb, and an electrical diffusion double layer occurred by Na(I), which caused repulsion between ions in the solution^50^. The Cd(II) adsorption capacities of Sp, Sa, Sb, and Ms were decreased by 57.0%, 68.5%, 62.5%, and 71.4%, respectively, at the ionic strength of 0.6 mol·L^−1^ compared with 0 mol·L^−1^. It could be seen that the adsorption of Cd(II) by Sa, Sb, and Ms was more susceptible to the inhibition due to the increase in ionic strength than that by Sp. The ionic strength-dependent adsorption indicated that cation exchange partly contributed to Cd(II) adsorption^54^. The presence of Na(I) increased the zeta potential (ζ), indicating the neutralization of deprotonated Si–O–sites^55^. The presence of Si in rice straw gave it an increased adsorption capacity for Cd(II) from wastewater^6^. Higher ionic strength tended to suppress Si release and further suppressed the adsorption^55^. For Sp, Sa, and Sb, the higher the ionic strength, the more the adsorption of Cd(II) on the adsorbent because of a higher portion of Si being inhibited as more Si–O–sites were deprotonated. Although the amount of Si contained in Ms was the smallest, the introduced free carboxyl groups from citric acid caused more H(I) to compete with Cd(II)^56^, so Ms was most effective in inhibiting Cd(II) adsorption.

## Conclusions

Different modification methods exerted different effects on the SSA, Si content, surface morphology, and O-containing functional group of rice straw, but the adsorption capacity for Cd(II) was increased with all the modified rice straw materials relative to the raw rice straw powder (Sp) control. Raising up the temperature (298~318 *K*), enhancing the initial Cd(II) concentration (0~800 mg·L^−1^), prolonging the contact time (0~1440 min), increasing the initial pH value (2~10), and reducing the ionic strength (0~0.6 mol·L^−1^) were all beneficial for increasing the adsorption of Cd(II) by the four adsorbents. rice straw ash, which had the largest SSA, possessed the largest adsorption capacity for Cd(II). Only the adsorption of Cd(II) by the rice straw biochar at 298 *K* was spontaneous, and surprisingly, all other adsorptions were nonspontaneous. The Cd(II) adsorptions by the four adsorbents were all chemical and were favorable, exothermic and order-increase processes. The pseudo-second-order model showed a strong fit for the adsorption kinetics of Cd(II) by the adsorbents. The adsorption capacity of Cd(II) by the adsorbents was less at low pH values and increased with the increase of initial pH in the solution. Due to higher Si content in rice straw ash and biochar and more free carboxyl group with citric acid modified rice straw, the inhibiting effect on Cd(II) adsorption due to the increase in ionic strength was greater under rice straw ash, rice straw biochar and citric acid modified rice straw than that with rice straw power. In our study, the optimum condition for Cd(II) adsorption by rice straw adsorbents turned out to be the combination of temperature at 318 *K*, initial Cd(II) concentration of 800 mg·L^−1^, contact time of 240 min, and no Na(I) interference. If more updated instruments, such as nitrogen adsorption-desorption and Transmission electron microscope, were used to examine Cd(II) adsorption, more interesting results would be obtained. Rice is a gramineous plant species with strong Cd(II) enrichment ability. Cd(II) is the most common toxic metal in paddy fields threatening safe rice supply. Future research needs to focus on the removal of Cd(II) from rice paddy fields *in situ* using local rice straw ash prepared by ashing with no incineration to reduce the pollution of Cd(II) in rice (both grains and straws) and thus resulting in safer rice production and healthier environment.
